# Service recommendation method based on text view and interaction view

**DOI:** 10.1038/s41598-025-96568-5

**Published:** 2025-04-05

**Authors:** Ting Yu, Yaqi Wang, Fangying Cheng, Tian Liang, Hongbing Liu

**Affiliations:** School of Information Engineering, Jiaxing Nanhu University, Jiaxing, People’s Republic of China

**Keywords:** Engineering, Mathematics and computing

## Abstract

With the increasing prosperity of web service-sharing platforms, more and more software developers are integrating and reusing Web services when developing applications. This approach not only meets the needs of developers but also is cost-effective and widely used in the field of software development. Usually, software developers can browse, evaluate, and select corresponding Web services from a web service-sharing platform to create various applications with rich functionality. However, a large number of candidate Web services have placed a heavy burden on the selection decisions of software developers. Existing web service recommendation systems often face two challenges. Firstly, developers discover services by inputting development requirements, but the user’s input is arbitrary and can not fully reflect the user’s intention. Secondly, the application service interaction record is too sparse, reaching 99.9%, making it particularly difficult to extract services that meet the requirements. To address the above challenges, in this paper, we propose a service recommendation method based on text and interaction views (SRTI). Firstly, SRTI employs graph neural network algorithms to deeply mine the historical records, extract the features of applications and services, and calculate their preferences. Secondly, SRTI uses Transformer to analysis develop requirements and uses fully connected neural networks to deeply mine the matching degree between candidate services and development requirements. Finally, we integrate the above two to obtain the final service list. Extensive experiments on real-world datasets have shown that SRTI outperforms several state-of-the-art methods in service recommendation.

## Introduction

With the development of Web 2.0, users are increasingly inclined to develop applications and create value through various services on the internet, such as product ordering, online chatting, etc. The number of Web services is showing a rapid growth trend, and enterprises such as Baidu and Google also publish Web services to users through methods such as service-oriented architecture (SOA)^1^ and service-oriented computing (SOC)^2^ to obtain additional economic benefits. At the same time, the development of Web 2.0 technology has provided technical support for the use of Web services, making them aimed at a wider user group rather than limited to a few professional users. The rapid development of big data, cloud computing, and artificial intelligence has provided a deployment platform for Web services. Any user can publish web service interfaces online and develop new value-added services. By calling and synthesizing these Web services, new applications can be generated.

Generally, service recommendation systems rely on the description requirements of the developers for historical invocation records to estimate the probability of services invoked by applications. Therefore, the existing works on service recommendation could be divide into two scenarios. One is based on the developer’s input text description, the other is based on the historical invocation records between applications and services. As for the first case, the works calculate the matching degree between the target application and candidate services. However, the text description provided by services’ documents is usually short, vague or inaccurate, resulting in low accuracy of such approaches. As for the second scenario, for the target application, several services have been selected already, the service recommendation system tries to recomend needed services. However, These approaches are more sensitive to data sparsity, which leads to a decrease in the recommendation accuracy.

In a real-world application development scenario, there are various types of information between applications and services, including explicit structural information, implicit structural information, explicit semantic information, and implicit semantic information. Explicit structural information refers to the structural information between applications and services that can be retrieved directly from repositories, e.g., invocation relationships. Implicit structural information refers to the structural information between applications and services that cannot be directly captured from the repositories but needs to be mined and inferred with techniques, e.g., collaboration and conflict relationships between services. Explicit semantic information refers to the semantic information of requirements and application or service descriptions without considering the relevance between contexts. Implicit semantic information refers to the latent semantic information of requirements and application or service descriptions by considering the relevance between contexts.

**Fig. 1:**
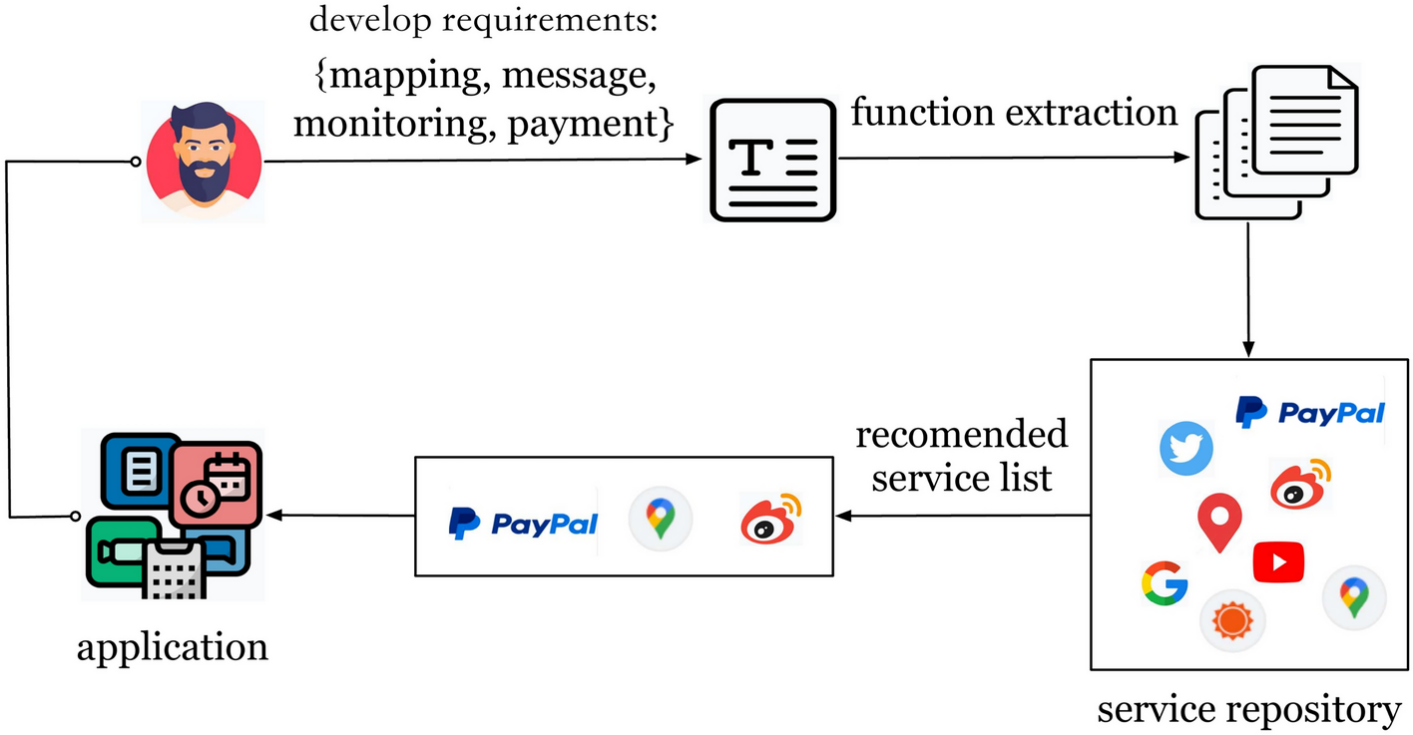
The process of service recommendation.

The large number of candidate services often makes it difficult for developers to make a decision, especially for those who do not have much background knowledge of the services. Moreover, It is difficult to fully mine and utilize various types of information between applications and services. For example, if a developer intends to complete an application with four functions {mapping, message, monitoring, payment}, as shown in Fig. 1, the developer first enters these four keywords and then searches for a set of candidate services from the service repository using the keywords, which means traversing a large number of services in the service repository^3–6^. Therefore, despite extensive research in this field, there are still the following challenges:

1. Users are not professionals in the service field and cannot accurately describe the functions of the required services.
2. There are a large number of services in the service repository, and the proportion of these services being invoked is very small. Most services are immersed in the repository, so how to accurately mine services that meet the requirements is another major challenge.

Considering the above challenges, we have improved the existing service recommendation method and proposed a Service Recommendation method based on Text and Interaction views (SRTI). The contribution of our paper is as follows:

1. We consider historical invocation records and utilize various auxiliary information to delve deeper into the expected functionality of the application to be developed.
2. We consider development requirements information and employ the Transformer model to explore the relationships between words.
3. We conducted extensive experiments on real-world datasets. The results demonstrate the effectiveness of our proposed method.

The remaining paper is structured as follows. “Related works” section introduces the related works. The proposed SRTI method is introduced in detail in “Service recommendation method based on text and interaction views” section. Afterwards, “Results and discussion” section gives the experimental results and disscussion. Finally, we summarize the paper and put forward our future work.

## Related works

In recent years, more and more researchers have devoted themselves to the development of application based on service recommendation from multiple perspectives. In this section, we summarize the progress of existing service recommendation work from three aspects: service recommendation based on development requirements, service recommendation based on collaborative filtering and service recommendation based on graph neural networks.

### Service recommendation based on develop requirements

The service recommendation method based on text requirements mainly extracts and matches features from the development requirements and service descriptions.

For example, Naim et al.^7^ combined probabilistic topic model and pattern mining to conduct in-depth analysis of service description documents and proposed a content-based semantic extraction method to obtain the maximum common semantics of the service set. However, it is challenging to determine the number of topics in the topic modelling of Web service. That is to say, the number of topics needs to be specified in advance before training to generate the implicit topic vector distribution of the description document. Meanwhile, it is necessary to adjust the number of topics to obtain the best service recommendation performance. To overcome some shortcomings of the topic model, Gu et al.^8^ used the topic model to build the semantic service pack repository and proposed a service pack recommendation model based on composite semantics. Besides, Pugazhenthi et al.^9^ used the ConceptNet knowledge graph to connect natural language words and phrases through the edges with labels (representing the type of edges) and weights (representing the credibility of edges) to enhance the data representation in the recommendation system and used mutual information maximization to align word level and entity level semantic spaces. Compared with existing methods, this method has strong language representation and feature extraction capabilities for text. In addition, Li et al.^10^ proposed an unsupervised Bayesian statistical model to realize semantic matching between possible text type queries (words, phrases, sentences, paragraphs) and texts in web service descriptions by mapping words and sentences in the same semantic space. Nguyen et al.^11^ proposed an attention probability matrix factorization model, which injects attention scores and development requirement context similarity into the matrix factorization structure for training to obtain a service recommendation list. In summation, the service recommendation based on develop requirements primarily focus on one-to-one relations only, i.e., application and service. They rely heavy on the text information, when the service description information is insufficient, the recommendation effect is not very satisfactory.

### Service recommendation based on collaborative filtering

Collaborative filtering based on the historical invocation application-service records used in the recommendation systems^12–14^. These method uses the historical records for analysis to find similar neighboor of applications or services.

In particular, Ren et al.^15^ proposed a location-aware collaborative filtering method that maps location features into a high-dimensional dense embedding vector to correct the prediction quality of the service. Their approach effectively improves the predictive quality of the service. However, the extracted intrinsic features are usually not related to the domain, which leads to poor recommendation effects. Considering this shortcoming, Cui et al.^16^ proposed a model based on the time correlation coefficient. Using the cuckoo search algorithm, they improved the Kmeans algorithm (CSK-means) to design an effective personalized recommendation model (PTCCF) based on preference patterns. Besides, Khelloufi et al. et al.^17^ proposed a service recommendation system based on the social relationship between device owners in which the recommendation was based on different relationships between service requesters and service providers. The experimental results showed that in the SIoT environment, incorporating users’ social relationships into service recommendations can improve the accuracy and diversity of provided services. Wang et al.^18^ proposed a user-trust-based collaborative filtering approach (TBCF), which combines User-Item-Trust (UIT) records with traditional CF-based models to improve the robustness of recommendations while maintaining recommendation accuracy. Bouazza et al.^19^ proposed an IoT recommendation system based on a hybrid technique (i.e., collaborative filtering and knowledgebased techniques). In particular, the authors proposed to use collaborative filtering and ontology to recommend IoT services that suit IoT services consumers’ needs. However, the above approach relies on the historical usage records between applications and services. Unfortunately, historical usage data is very sparse. Most applications use no more than three APIs, and only 10% of the existing services are used by applications, which makes it difficult to recommend appropriate services when the scale of data is very large.

### Service recommendation based on graph neural networks

With the rapid development of graph neural networks, applying various graph neural network technologies to service recommendation has become a trend. Unlike methods based on text requirements, methods based on graph neural networks mine information from historical interactions between services or requirements (users) to make recommendations.

For example, inspired by Zhu et al.^20^, the bilinear graph neural network was introduced into web service classification, and the multiplication-based bilinear news aggregator was used to capture the interaction between service nodes in the neighborhood. Then, combined with multidimensional QoS attribute information, the XDeepFM model^21^ is used to integrate explicit higher-order interaction modules, implicit interaction modules, and traditional FM modules. In the above methods, graph neural networks can effectively solve the problem of data sparsity in service classification methods based on functional semantics. However, the existing graph neural network methods ignore the possible interaction information between adjacent service nodes. When such interaction exists, extracting the interaction information can further enhance the representation of the target service node. Therefore, Wang et al.^5^ proposed a knowledge graph-based deep random walk unsupervised service recommendation method, which comprehensively considers the service domain and the correlation between development requirements and services. By designing an entity biased random walk process, the development requirements’ preferences for each service are dynamically adjusted. Besides, Shi et al.^22^ proposed a two-step service graph convolutional network model. In the first step, predict the potential connection between APIs and mashups based on the similarity of text content. Based on the common calling relationship between mashups and APIs, edges were added between the two, and an attention-based RTM (Attention RTM) model was trained for web service link prediction. The second step is to apply two GCN layers with ReLU activation function to learn node features for Web services.

Moreover, Recent advancements in recommender systems have addressed challenges in federated and adversarial settings. For example, Yuan et al.^23,24^ investigated interaction-level membership inference attacks and poisoning attacks using synthetic users, highlighting privacy risks and proposing countermeasures. Heterogeneity in federated systems has also been explored^25^. On the adversarial front, Chen et al.^26^ studied manipulation of visually-aware systems through adversarial item promotion. Additionally, the integration of large language models (LLMs) into federated systems has been examined^27^.

## Service recommendation method based on text and interaction views

In this section, we formulate the service recommendation problem and related key concepts. Table 1 shows the main symbols and their explanations.

**Table 1:** Symbols used in this work.

Symbol	Description
*S*	Service set
*s*	A service
$s_{name}$	The name of service s
$s_{des}$	The description of service s
$s_t$=($s_{t_1},s_{t_2},\ldots ,s_{t_n}$)	The tags of service *s*
*ST*	The tag set of *S*
*A*	Application set
*a*	An application
$a_{name}$	The name of application *a*
$a_{des}$	The description of application *a*
$a_t$=($a_{t_1},a_{t_2},\ldots ,a_{t_m}$)	The tags of application *a*
*AT*	The tag set of *A*

### Definition 1

(*Service*) A service *s* is a collection of publicly accessable functions, formalized as a 3-tuple, i.e., $s=<s_{name};s_{des};(s_{t_1},s_{t_2},\ldots ,s_{t_n})>$, where $s_{name}$, $s_{des}$, $(s_{t_1},t_{t_2},\ldots ,s_{t_n})$ represent the name, text description and tag sets of *s*, respectively.

### Definition 2

(*Application*) Among various realizations of service composition, applications represent the data-centric, lightweight Web applications that are usually designed and developed in an agile, visual, and interactive manner. An application implies easy, fast integration of Web APIs (i.e., Web services) to satisfy a broader set of requirements, collectively realizing new functionality.

An application *a* is the composition of two or more services to provide functionality or commercial value, formalized as a 4-tuple, i.e., $a=<a_{name}; a_{des};(s_1,s_2\ldots s_n);(a_{t_1},a_{t_2},\ldots ,a_{t_m})>$, where $a_{name}$, $a_{des}$, $(a_{t_1},a_{t_2},\ldots ,a_{t_m})$ represent the name, text description and tag sets of *a* and $(s_1,s_2\ldots s_n)$ represents the services invoked by *a*. Applications frequently act as a bridge between a diverse set of services given by different providers.

### Definition 3

(*Service repository*) All the services from the service set $S=\{s_1,s_2,\ldots ,s_p\}$. All the applications constitute a set $A=\{a_1,a_2,\ldots ,a_q\}$. A service repository is the union of *S* and *A*, i.e., $R=S\cup A$.

Table 2 shows an example of an application and a service from the data.

**Table 2:** An example of an application and a service.

Application: EasyWeather		Service: Instagram graph API	
Attribute	Value	Attribute	Value
Name	EasyWeather	Name	Instagram Graph API
Tag	Web	Tag	Photos, Mobile, Social
Description	EasyWeather is a lightweight and versatile jQuery plugin displaying weather information for any location	description	Instagram is a photo sharing iPhone app and service. Users take photos and can share them with Instagram contacts, as well as friends on other social networks like Twitter and Facebook
Invoked services	World Weather Online, Yahoo Weather, OpenWeatherMap, Weather Underground, Forecast		

### Definition 4

(*Application-service invocation records*) Give the application set $A=\{a_1,a_2,\ldots a_p\}$ and the service set $S=\{s_1,s_2,\ldots s_q\}$, application service invocation matrix $Y\in R^{p\times q}$ is defined based on the application service invocation records. For example, application *a* and service *s*, when $y_ {(a, s)}=1$ means that *s* has been invoked by *a*, otherwise, $y_ {(a, s)}=0$.

**Problem Definition:** *Given the develop requirements* $a'_{des}$ *of the application* *a* *and the previous invocation records between application set* *A* *and services set* *S*, *for an application* $a'=<a'_{name}; a'_{des}; (s_1,s_2\ldots s_p); (a'_{t_1},a'_{t_2},\ldots ,a'_{t_m})>$, *the problem to be addressed is to find the Top-K services, i.e.,* $(s_{p+1},s_{p+2}\ldots s_{p+k})$ *for* $a'$ *in* *S*, *which are most likely to be invoked by* $a'$.

### Framework

**Fig. 2:**
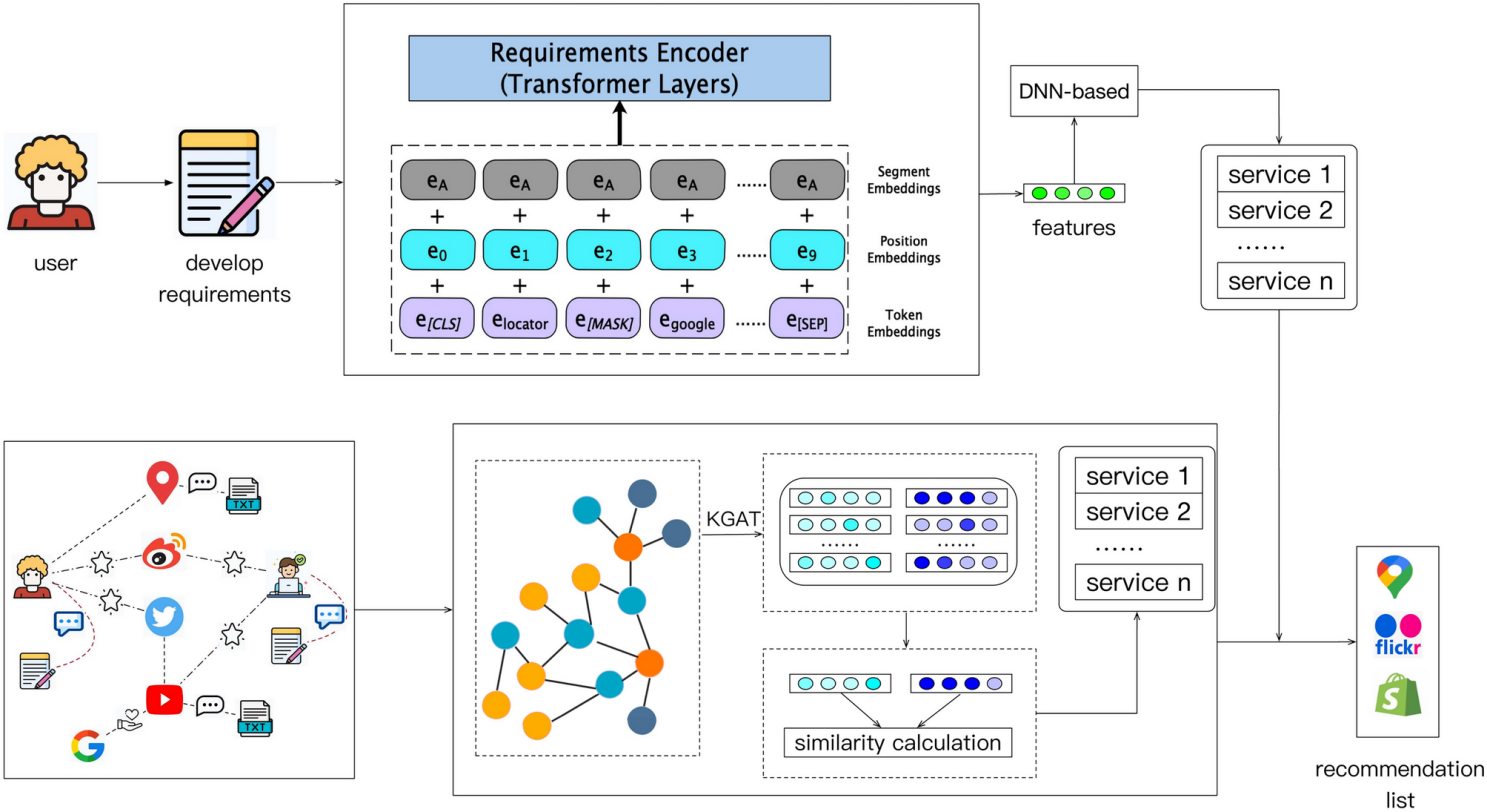
The framework of SRTI.

Figure 2 shows the overall framework of SRTI proposed in this article. Firstly, the Transformer model is used to extract the characteristics of application development requirements. Then, a deep neural network is used to deeply mine the matching degree between candidate services and development intentions, and the application’s text preferences for each service can then be obtained. Secondly, the application-service-tag network graph is obtained through the application service history invocation records. The KGAT model is employed to deeply mine the application-service invocation information, extract the application and service features, and obtain the structural preferences of the application for each service. Finally, integrate the above two to obtain the final service recommendation list.

### Service recommendation based on text View

**Fig. 3:**
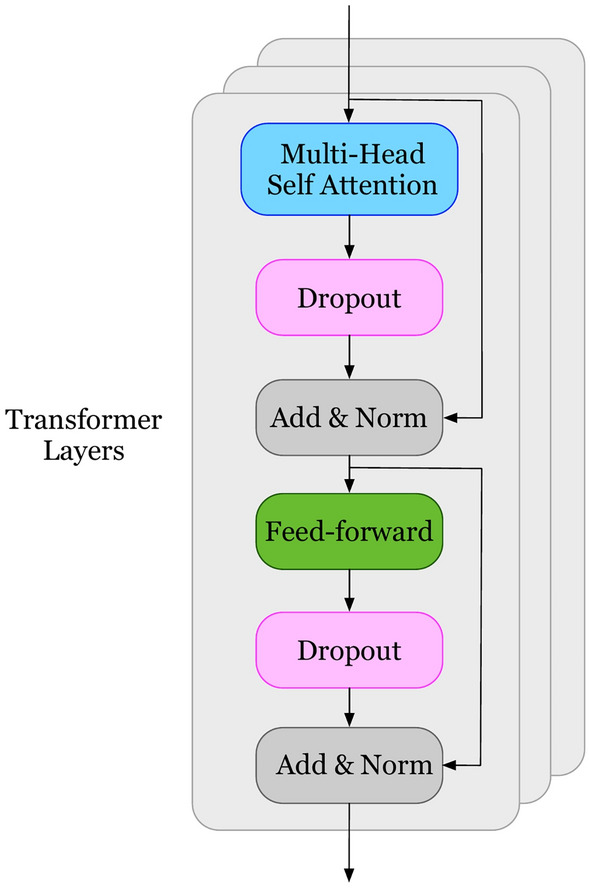
The framework of transformer.

Since the Transformer^28^ model was proposed, tattention-basedased neural network has achieved tremendous success in many fields. The encoder part of Transformer has a strong ability to learn the interactive information between sequence elements. Inspired by this, we use a Transformer encoder to extract features from text, enabling the model to better understand the development requirements. The framework of Transformer as shown in Fig. 3.

Given the development requirements $a_{des}$, the Transformer encoder extracts feature representations for all words, and the output can be expressed as $vec_{a_{des}}=\{vec_1,vec_2,\ldots ,vec_L\}$. Where *L* represents the length of development requirements, $vec_i$ represents the representation of the word in position *i*.

The encoder consists of *N* identical layers, each containing two sub layers, namely self-attention mechanism and fully connected feedforward network. Assuming the input is *g*, the sub layers apply residual connections, and then perform layer normalization to calculate the output, which can be represented as $LayerNorm(g+SubLayer(g))$, where *SubLayer* represents a self-attention mechanism and a fully connected feedforward network.

Since each layer of the encoder is the same, the *l*th layer is selected for a brief introduction. The input of layer *l* is the output of the previous layer. The input of the first layer is an embedding representation of the document requirements $vec_{a_{des}}=X$. In the self-attention mechanism, query *Q*, keyword *K*, and value *V* are all linear projections of $vec_{a_{des}}^{l-1}$ with different parameter matrices. The output of self-attention is calculated based on scaled dot product attention, and the calculation process is as follows:

1 $$\begin{aligned} Att(Q,K,V)=Softmax\left( \frac{Q^TK}{\sqrt{d_e}}\right) V \end{aligned}$$

In addition, the Transformer encoder implements self-attention by parallel computing the *h* heads, where each head calculates attention based on Eq. (1). The output of multi-head attention is a concatenation of *h* heads, followed by linear projection, as follows:

2 $$\begin{aligned} \begin{aligned} MultiHead(Q,K,V)=f(H_1,H_2,\ldots H_h)W^M \\ H_i=ATT(QW_i^Q,KW_i^K,VW_i^V) \end{aligned} \end{aligned}$$

where *f* represents the concatenate operation, $W^M, W_i^Q, W_i^K, W_i^V$ represents the parameter matrix. After the self-attention mechanism sublayer, the fully connected sublayer obtains the output of self-attention, represented as follows:

3 $$\begin{aligned} FFN(X_l)=ReLU(E_{ATT}^lW_1+b_1)W_2+b_2 \end{aligned}$$

where $W_1, W_2, b_1, b_2$ represents trainable parameters. After N-layer encoding, the Transformer encoder obtains the develop requirements representation $vec_{a_{des}}$. Then $vec_{a_{des}}$ is fed into the DNN layer to further extract non-linear interactions of text features. This process can be expressed as:

4 $$\begin{aligned} vf_{a_{des}}=W_i(vec_{a_{des}})+b_i \end{aligned}$$

Finally, the learned vector $vf_{a_{des}}$ is input into the sigmoid function and the output is $y_{a,s}[a,s]$. $y_{a,s}[a,s]$ represents the probability of service *s* being selected by application *a*, and this process is defined as follows:

5 $$\begin{aligned} y_{a,s}[a,s]=sigmoid(W_{a,s}vf_{a_{des}})+b_{a,s} \end{aligned}$$

where $W_{a,s}, b_{a,s}$ represents the weight matrix and bias.

In order to train the model task, binary Cross entropy loss (BCELoss) is adopted, which is defined as follows:

6 $$\begin{aligned} L_{a,s}=\frac{1}{\vert S \vert }\sum _{s\in S}(y_{a,s}[a,s]log\hat{y}_{a,s}[a,s]+(1-y_{a,s})log(1-\hat{y}_{a,s}))+\lambda _1\Vert \Theta \Vert _2^2 \end{aligned}$$

where $y_{a,s}[a,s]$ represents whether application *a* has actually invoked service *s*. If application *a* has invoked service *s*, then $y_{a,s}[a,s]=1$, otherwise, $y_{a,s}[a,s]=0$. $\hat{y}_{a,s}[a,s]$ represents the probability of service *s* being selected by application *a*.

### Service recommendation based on interaction view

**Fig. 4:**
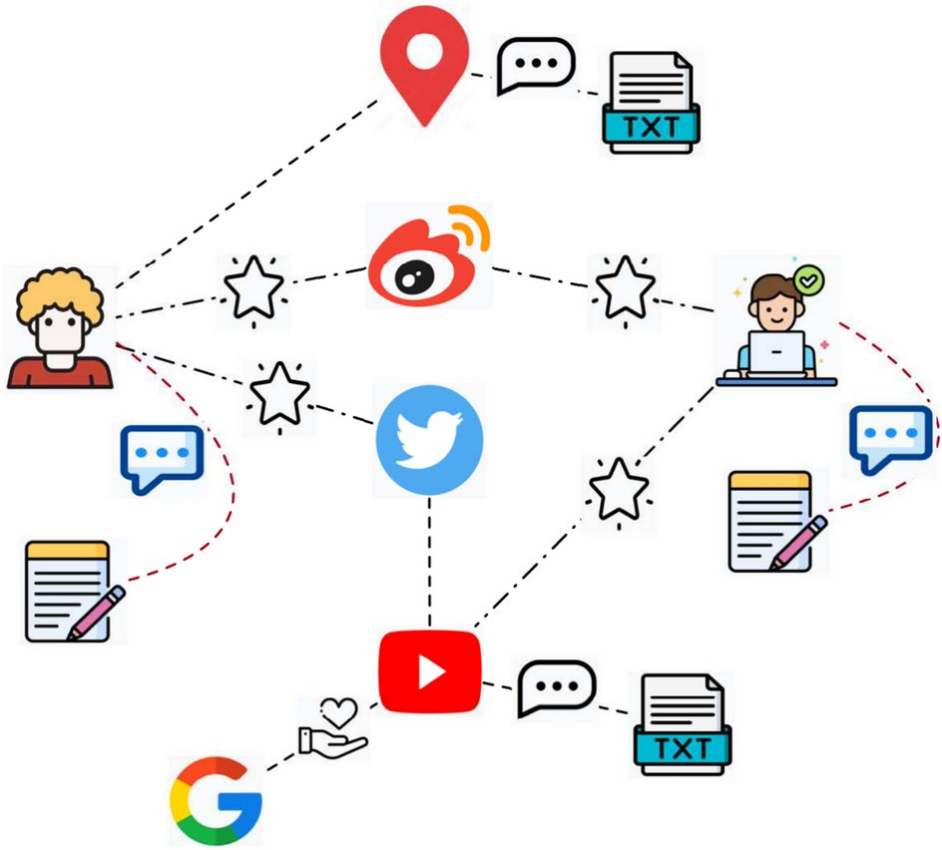
The example of application-service-tag graph.

Based on the historical invocation records, an application-service-tag network graph $G=(V,E)$ can be constructed, where $V=\{v_1,v_2,\ldots ,v_z\}$ represents the node set, and $E=\{e_{i,j}=1\}_{i,j=1}^Z=E_{a,s}\cup E_{s,t}\cup E_{a,t}$ represents the set of edges. The application set *A*, service set *S*, and tag set *T* are used to construct the node set $V=A\cup S\cup T$. For edge set *E*, if there is an invocation between application *a* and service *s*, they are connected in the graph, thus forming edge set $E_{a,s}$, and if tag *t* belongs to a certain service *s*, then these two are connected in the graph, forming an edge set $E_{s,t}$. Similarly, if tag *t* belongs to an application *a*, then the two are connected in the graph, forming an edge set $E_{a,t}$. Figure 4 shows an example of a application-service-tag graph.

**Fig. 5:**
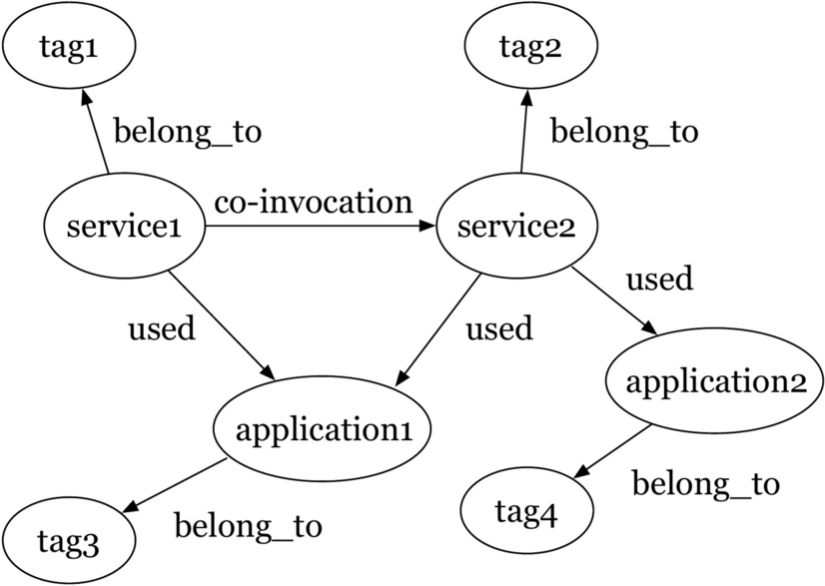
Relations in the knowlege graph.

After constructing the application service heterogeneous graph, we build a service knowledge graph, as shown in Fig. 5. As can be seen from Fig. 5. The relation between service and application was “used”, and the relation between service and tag was “belong_to”. In addition, the relation between application and tag is “belong_to”, and if two services have been invoked by the same application, then the relation between the two services is “co-invocation”.

Then we employ the graph neural network KGAT^29^ to extract historical application and service representations. KGAT mainly consists of three parts, namely the embedding layer, attention propagation layer, and feature representation generation. Finally, the feature representation of applications and services can be obtained through KGAT.

(1) Embedding layer

The embedding layer is a module used to obtain vector representations of entities and relationships in a knowledge graph. KGAT uses TransR^30^ to embed entities and relationships in the knowledge graph. Given the graph *G*, for a triplet $(h,r,t)\in G$, the calculation process is as follows:

7 $$\begin{aligned} En(h,r,t)=\Vert W_re_h+e_r+W_re_r\Vert _2^2 \end{aligned}$$

where $W_r\in R^{k\times d}$ is a transformation matrix of relationship *r*. For triplets (*h*, *r*, *t*), *h* and *t* represent applications or services, and *r* represents their relationships in the knowledge graph. Its loss function is defined as follows:

8 $$\begin{aligned} L=\sum _{(h,r,t,t')}-ln\sigma (En(h,r,t')-En(h,r,t)) \end{aligned}$$

where $T=\{(h,r,t,t')|((h,r,t)\in G,(h,r,t')\notin G)\}$. $(h,r,t')$ is a triplet constructed by randomly replacing an entity in a valid triplet. $\sigma (\cdot )$ indicates the activation function.

(2) Attention transportation layer

The attention propagation layer transfers information from neighboring nodes to target nodes to capture the interaction patterns between nodes. It is mainly divided into three parts: information propagation, knowledge perception attention, and information aggregation. Knowledge dissemination models the propagation of information from one entity to another, with all entities being influenced by the surrounding entities in the graph network structure. To represent first-order connections of entities, KGAT uses the ego network^31^. Knowledge perception attention utilizes relational attention mechanisms to achieve communication between each pair of entities. Information aggregation aggregates entity representations and their ego network representations into new representations. At this stage, three types of aggregations are used, namely GCN aggregator, the GraphSage aggregator, and Bi-Interaction aggregator. Then, propagate to multiple layers to explore higher-order connectivity information.

(3) Feature representation generation

After performing L-order propagation, for application node *a* and service node *s*, the overall characteristics are represented as follows:

9 $$\begin{aligned} e_a^*= & e_a^0\Vert e_a^1\Vert \cdots \Vert e_a^L \end{aligned}$$

10 $$\begin{aligned} e_s^*= & e_s^0\Vert e_s^1\Vert \cdots \Vert e_s^L \end{aligned}$$

Where $\Vert$ represents the concatenate operation.

Finally, the preference of application *a* for service *s* can be expressed as:

11 $$\begin{aligned} sim_{a,s}=e_a^{*T}\cdot a_s^* \end{aligned}$$

### Model fusion

Considering both textual information and historical invocation records, we can obtain two preferences, the final service recommendation can be obtained as follows:

12 $$\begin{aligned} p_a^s=(1-\alpha )*sim_{a,s}+\alpha *\hat{y}_{a,s}[a,s] \end{aligned}$$

where $0\le \alpha \le 1$, $\alpha$ represents the importance of service preferences obtained based on application history invocation records.

All services in the candidate service set are sorted according to to their $p_a^s$. The result will be provided to developers with supplementary information, such as the description of services. For example, if a developer enters a requirement description as let users virtually explore different destinations around the world, Depending on the destination, the web application supplies pictures and information on the historical background, significance, popularity, and more. The approach will generate a list containing three dimensions such as Top-K sequence, service name, service description, etc. Top-K indicates the recommended order of services. A sample of the recommendation result is shown in Table 3.

**Table 3:** A sample recommendation result.

Top-K	Service name	Serivce description
1	Facebook	The API allows applications to use the social connections and profile information $\ldots$
2	Flickr	The Flickr API can be used to retrieve photos from the Flickr photo sharing service $\ldots$
3	GeoNames	The GeoNames covers all countries and contains over eight million placenames $\ldots$
4	Google Maps	The Google Maps API allow for the embedding of Google Maps onto web pages $\ldots$
5	Google Geocoding	Geocoding API provides a direct way to access a geocoder via an HTTP request $\ldots$
$\ldots$	$\ldots$	$\ldots$

The algorithm for SRTI is as follows.

**Algorithm 1:**
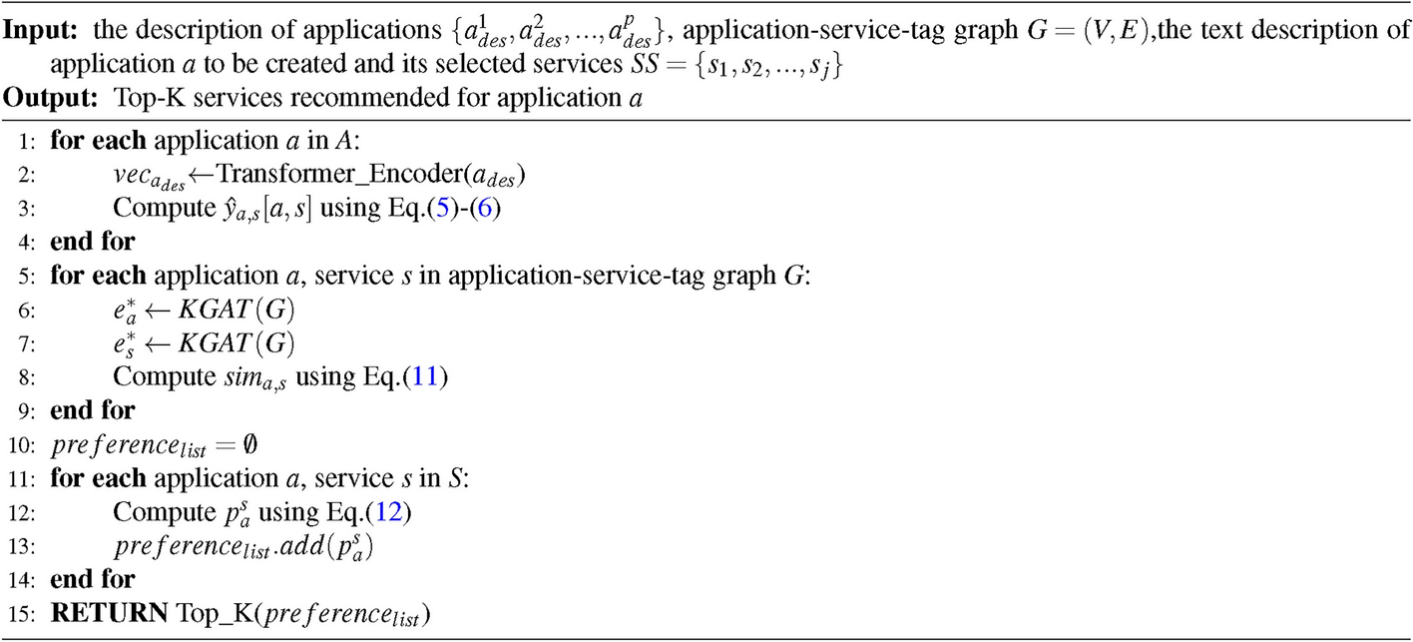
The algorithm of SRTI.

## Results and discussion

In this section, we evaluated SRTI performance on real-world datasets. Specifically, the experiment aims to answer the following three research questions:

- **RQ1:** Is SRTI better than other comparison methods in service recommendation tasks?
- **RQ2:** Can the text view and interaction view in the SRTI method capture information effectively?
- **RQ3:** How do the parameters in SRTI affect the recommendation results?

All experiments were developed in Python and conducted on a personal computer equipped with a 2.4GHz Intel Core i5 CPU, 8 GB RAM, and macOS High Sierra.

### Dataset

We have crawled 22,016 services and 6438 applications from ProgrammableWeb, the world’s largest online Web service registry. Applications and services without function descriptions, services without invoked, and applications with fewer than three component services are deleted from the original dataset. The final experimental dataset contains 6347 applications and 1623 services, the statistics of the dataset is shown in Table 4. The sparsity of the application service invocation matrix is 99.87%. We randomly divide the whole data set into five parts, use one of them as the test set, and combine the other four parts as the training set. We run the algorithm five times, and finally take the average value of the five times as the final result.

**Table 4:** Statistics of the experimental dataset.

	Value
#Applications	6437
#Services	1623
#Application tags	416
#Service tags	365
#Services per application	2.03
#Tags per application	2.93
#Tags per service	2.40
#Sparsity	99.9%

### Evaluation metric

**Fig. 6:**
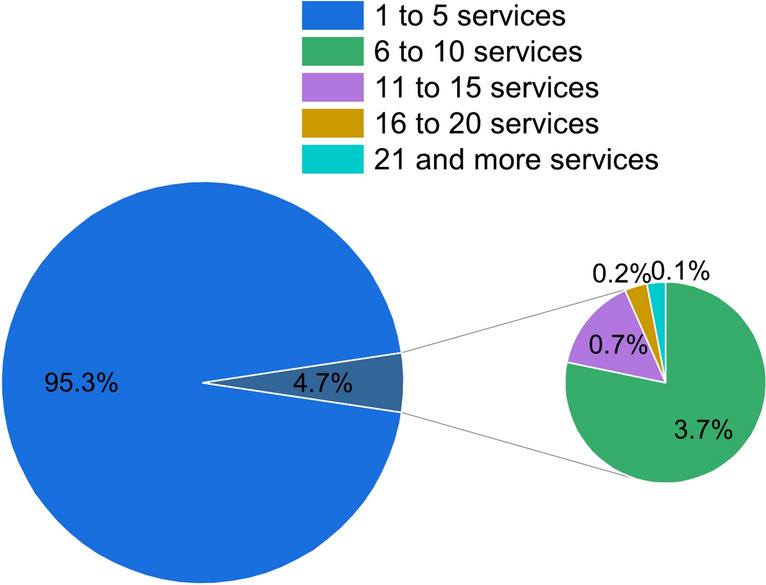
The distribution of the number of services invoked by applications.

Figure 6 shows the distribution of the number of services invoked by applications in the dataset. It can be seen that more than 95.3% of applications invoke less than five services. Therefore, the variable K in the Top-K services was set from 1 to 5.

We measured the results by *Precision*@*K*, *Recall*@*K* and *NDCG*@*k* discussed as follows.

13 $$\begin{aligned} Precision@k=\frac{1}{n}\sum _{a=1}^{n}\frac{|top_a(k) \cap test_a|}{k} \end{aligned}$$

The data in the training set is usually divided into positive samples and negative samples. In specific examples, positive samples refer to the services invoked by the application, while negative samples refer to the services not invoked by the application. Precision@k represents the proportion of predicted positive samples that are actually positive. As shown in Eq. (13), where $top_a(k)$ represents the top *k* services recommended to the application *a*, and $test_a$ represents the services actually invoked by application *a* in the test set.

14 $$\begin{aligned} Recall@k=\frac{1}{n}\sum _{a=1}^{n}\frac{|top_a(k) \cap test_a|}{|test_a|} \end{aligned}$$

Recall@k represents the proportion of predicted positive samples in the actual positive sample.

15 $$\begin{aligned} DCG@k= & \sum _{i=1}^k \frac{2^{rel_i}-1}{log_2(1+i)} \end{aligned}$$

16 $$\begin{aligned} NDCG@k= & \frac{DCG@k}{IDCG@k} \end{aligned}$$

Normalized Discounted Cummulative Gain (*NDCG*) is a measure of accuracy from information retrieval as shown in Eq. (16), *DCG* is accumulated from the top of the result list to the bottom, with the gain of each result discounted at lower ranks.

### Baselines

The comparison methods selected in the study are as follows:

- FISM^32^: FISM is a service-based method and it learns the similarity among services in latent factors, which aims to improve the predefined similarity in neighborhood-based methods for one-class collaborative filtering.
- SPR^33^: The SPR method utilizes application description and call information to mine important lexical features of services, further bridging the lexical gap between application developers and service providers. At the same time, it uses the Author Topic Model (ATM) to reconstruct the service configuration file.
- APR^34^: APR is an adversarial personalized ranking method based on BPR. It includes an additional objective function to boost performance.
- LightGCN^35^: LightGCN is a recommendation method based on simplified and enhanced graph convolutional networks, which only includes the most important components of GCN. The node representation can be obtained by linearly propagating the embedding of applications and services on the application service interaction graph, and finally use the weighted sum of application and service embedding as the final prediction score.
- SGL^36^: SGL augments data using random walk and feature dropout to generate multiple views. It enhances LightGCN with self-supervised contrastive learning.
- MTFM^37^: MTFM utilizes semantic components to generate requirement representations and introduces feature interaction components to model feature interactions between applications and services, thereby generating service lists.

### Performance (RQ1)

**Fig. 7:**
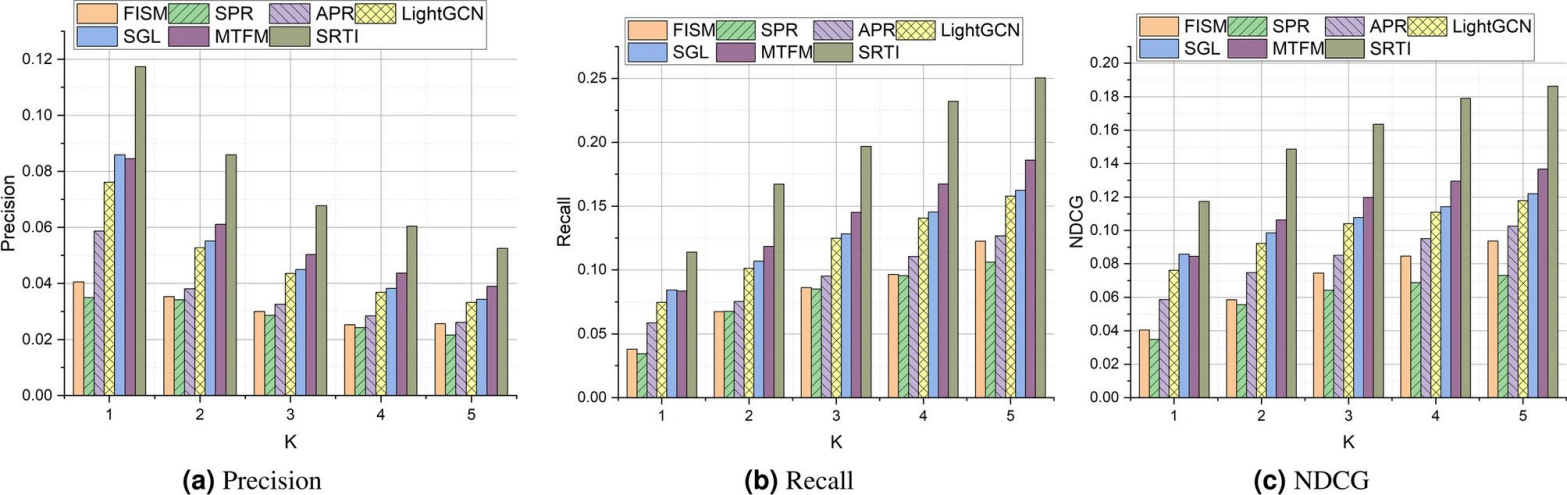
Performance comparison of different methods.

Figure 7 shows the performance comparison of different methods in terms of accuracy and recall. Due to the low density of application-service invocation records, the overall performance of indicators such as service recommendation accuracy is not high. As shown in Fig. 7, the methods such as FISM, SPR, and APR do not perform well in terms of accuracy or recall. The SPR method is superior to FISM in that it reconstructs and characterizes user profiles using application descriptions and contextual information that may affect user selection of Web services. LightGCN performs better than APR, because it uses graph convolution to obtain features and aggregates the features of neighbor nodes. SGL employs contrastive learning to learn representations of applications and services and performs better than LightGCN. MTFM comprehensively considers the historical invocation records and the text information, and it also introduces multi-task learning to recommend services. However, the matrix factorization method in MTFM is also limited by the influence of data sparsity. The dataset used in this paper is very sparse, reaching 99.9%. Our proposed method achieved the best results, which to some extent reveals that our method can effectively handle the problems caused by data sparsity and unsatisfactory text.

### Ablation experiments (RQ2)

**Fig. 8:**
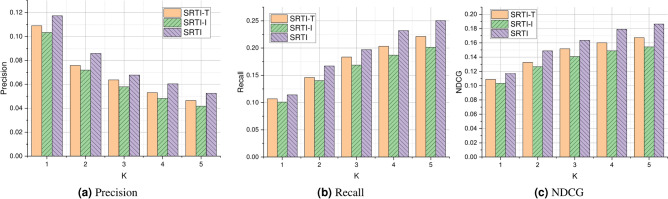
Performance of SRTI variants.

When recommending services for applications, the SRTI method not only analyzes the textual information, but also takes into account historical service invocation record information. In order to prove the necessity of considering the above factors, two variants of SRTI were designed, namely SRTI-T and SRTI-I. SRTI-T is obtained by removing development requirements, and SRTI-I is obtained by removing historical invocation record information. As shown in Fig. 8: (1) Among all variants, the SRTI method has the best performance, which proves the effectiveness of comprehensively considering development requirements and recording historical invocation records. (2) The suboptimal performance of SRTI-T indicates that considering historical invocation records is crucial for recommendation. (3) SRTI-I has the worst performance, and service recommendation should not only consider the textual information of the service.(4) From the experimental results, it can be seen that the experimental results of SRTI-I and SRTI-T are still superior to the comparative methods. Overall, the SRTI method provides an effective solution for creating application recommendation services.

### Parameter analysis (RQ3)

**Fig. 9:**
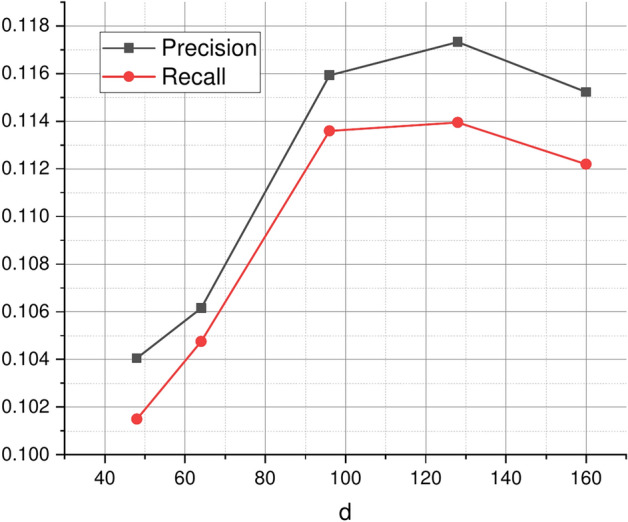
Recommendation performance with *d*.

The impact of dimension *d*. By changing $d=\{48,64,\ldots ,160\}$, the effect of the length of potential features on model performance is studied. When k=1, the values of Precision and NDCG are the same, so only Precision is displayed here. Figure 9 shows how the effects of various indicators on service recommendation change as *d* increases. As shown Fig. 9, the value gradually increases with the increase of *d*. However, when $d=128$, the metric value reaches its highest value and gradually decreases as *d* increases. As can be seen from Fig. 9, it is intuitive that having too few potential features (for example, dimension d = 10) limits the model’s ability to extract relevant information, thus leading to poor performance. However, an excessive number of potential features will lead to overtting, which reduces the performance of the model.

**Fig. 10:**
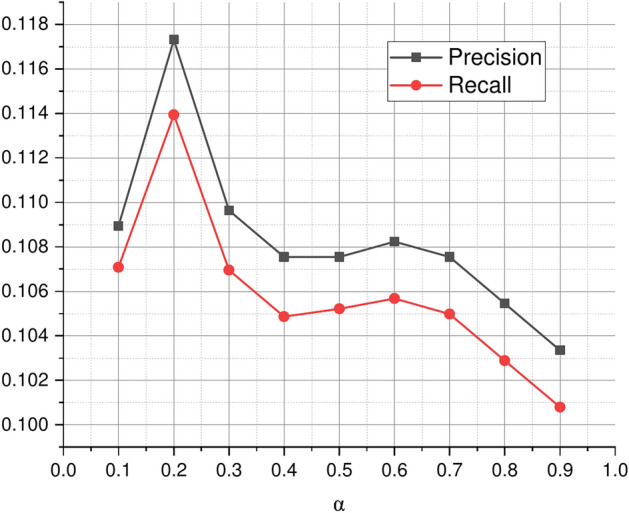
Recommendation performance with $\alpha$.

The impact of parameter $\alpha$. By changing $\alpha =\{0.1,0.2,\ldots ,0.9\}$, the performance on service recommendation was studied. $\alpha$ represents the importance of application’s preferences for services learned from invocation records. It can be seen that both invocation records and text information make contribution to service recommendation, however, the degrees of contribution of invocation records and text information to the service recommendation results are different. As shown in Fig. 10, when the alpha $\alpha$ equals to 0.7, the performance is best.

## Threats to validity

### Threats to internal validity

The threat to internal validity is related to the parameter selection and implementation of SRTI and other baselines. We froze the mode once all parameters were set in the validation set and then used an independent test set to obtain the final experimental results. For the baseline approaches, we made some modifications based on their published open-source code and adapted it to the input and output formats required by the experiments. During the modification process, each line of code was developed and reviewed by an author. All authors have rich experience in code development, so the implementation of all approaches was basically non-threatening.

### Threats to external validity

The threat of external validity is related to the generalizability of the approach. Currently, SRTI is only evaluated on the PWeb dataset, which can be a potential threat due to the missing documentation of some services and applications or the lack of usage records for some services. This disables the dataset from encompassing all services and applications on the PWeb. In the future, we plan to obtain more services and applications from other publicly available sites to enrich our dataset. In addition, due to operational policy changes, the PWeb website shut down. down its services, which prevented us from further expanding and updating the dataset from it.

## Conclusions

The reuse of Web services provides an effective method for software developers to quickly and economically create applications. However, traditional keyword-based web service recommendation methods impose a heavy burden on developers in making service selection decisions, especially when they lack any professional background knowledge. Considering these challenges, in this paper, we propose a Service Recommendation method based on Text and Interaction views (SRTI). In SRTI, we comprehensively consider the textual information and historical invocation records of development requirements. Finally, we validated the effectiveness of SRTI through a set of real data experiments from ProgrammableWeb. Diversity can also affect developer satisfaction. In future research, we plan to explore long tail services to improve the diversity of service recommendations and developer satisfaction. In addition, we plan to introduce more dimensions in SRTI, such as service privacy, time cost, etc. SRTI was evaluated on the ProgrammableWeb dataset due to its relevance and availability for the specific task at hand. In future work, we plan to evaluate SRTI on additional datasets from different domains to better understand its performance in varied contexts. Future research will focus more on validating the SRTI method in practical situations to ensure its relevance and effectiveness in real-world scenarios.

## Data Availability

The datasets used and generated during the current study are available from the corresponding author upon reasonable request.
